# Critical gaps in understanding firearm suicide in Hispanic communities: demographics, mental health, and access to care

**DOI:** 10.1093/haschl/qxad016

**Published:** 2023-06-20

**Authors:** Evan V Goldstein, Francisco Brenes, Fernando A Wilson

**Affiliations:** Department of Population Health Sciences, Spencer Fox Eccles School of Medicine, University of Utah, Salt Lake City, UT 84108, United States; Nicole Wertheim College of Nursing and Health Sciences, Florida International University, Miami, FL 33199, United States; Department of Population Health Sciences, Spencer Fox Eccles School of Medicine, University of Utah, Salt Lake City, UT 84108, United States; Department of Economics, University of Utah, Salt Lake City, UT 84112, United States; Matheson Center for Health Care Studies, University of Utah, Salt Lake City, UT 84112, United States

**Keywords:** firearms, suicide, Hispanic/Latino health

## Abstract

Suicide rates increased by 26.7% among Hispanics from 2015 to 2020, driven at least in part by highly lethal firearm suicide deaths. However, there are critical gaps in characterizing firearm suicide risks and prevention opportunities in Hispanic communities. We examined Hispanic adult firearm suicide decedents reported through the National Violent Death Reporting System from 2013–2019, focusing on demographic characteristics, firearm choices, suicidal thoughts/behaviors, mental health, and mental health treatment, compared with non-Hispanic adult firearm suicide decedents. Only 13.8% of Hispanic firearm suicide decedents were known to be undergoing treatment for a mental health or substance use problem prior to death, compared to 18.8% of non-Hispanic firearm suicide decedents. On average, Hispanic firearm suicide decedents were significantly less likely than non-Hispanic firearm suicide decedents known to have been treated for a mental health or substance use problem. These results may underscore the critical need for public health agencies and policymakers to promote initiatives integrating mental health screening into medical care, reducing mental health stigma among Hispanics, and expanding mental health treatment capacity in Hispanic communities.

## Lay Summary

We examined Hispanic adult firearm suicide decedents reported through the National Violent Death Reporting System from 2013–2019, focusing on demographic characteristics, firearm choices, suicidal thoughts and behaviors, mental health, and mental health treatment, compared with non-Hispanic adult firearm suicide decedents. We found key differences in demographic characteristics, firearm choices, and known history of suicidal thoughts and behaviors, mental ill health, and mental health treatment between Hispanic and non-Hispanic adult firearm suicide decedents. One key finding from this study was that Hispanic adult firearm suicide decedents were significantly less likely than non-Hispanic adult firearm suicide decedents known to be undergoing treatment for mental health or substance use problems prior to death. Additionally, compared with non-Hispanic decedents, Hispanic decedents were more likely to have a known history of suicidal thoughts/plans and suicide attempts. Handguns were used in three-fourths (74.9%) of non-Hispanic firearm suicide deaths but more than 8 in 10 (81.9%) Hispanic firearm suicide deaths. These results may indicate opportunities for public health agencies and policymakers to promote initiatives integrating mental health screening into primary medical care, reducing mental health stigma among Hispanics, and expanding mental health treatment capacity in Hispanic communities.

## Teaser

Suicide is one of the leading causes of death in the United States, and over 40 000 lives have been lost to suicide in each of the last 9 years. Suicide deaths are becoming more common in communities of color. Suicide rates increased by 26.7% among Hispanics from 2015 to 2020, driven at least in part by highly lethal firearm suicide deaths. However, there are critical gaps in characterizing firearm suicide risks and prevention opportunities in Hispanic communities. By examining all adult firearm suicide deaths reported in the National Violent Death Reporting System from 2013–2019, we were able to demonstrate key differences in demographic characteristics, firearm choices, and known history of suicidal thoughts and behaviors, mental ill health, and mental health treatment between Hispanic and non-Hispanic decedents. One key finding from this study was that Hispanic adult firearm suicide decedents were significantly less likely than non-Hispanic adult firearm suicide decedents known to be undergoing treatment for mental health or substance use problems prior to death. These results indicate opportunities for public health agencies and policymakers to promote initiatives integrating mental health screening into primary medical care, reducing mental health stigma among Hispanics, and expanding mental health treatment capacity in Hispanic communities.

## Introduction

Suicide is one of the leading causes of death in the United States, and over 40 000 lives have been lost to suicide in each of the last 9 years.^1^ Suicide deaths are becoming more common in communities of color.^2^ Among Hispanics,^3^ suicide rates increased 26.7% from 2015 to 2020 (from 5.89 per 100 000 persons to 7.46 per 100 000 persons),^1^ compared to a less than 0.5% increase in non-Hispanic suicide rates over the same period. Suicidal thoughts and behaviors are complex, polygenic phenotypes and vary by population.^4^ Suicide risks among Hispanics can vary across nationalities and US- and foreign-born populations,^5,6^ as do lifetime suicidality rates.^7^ Specific sociocultural and environmental factors can also increase the likelihood of suicidality, mental ill health, and psychological distress in different Hispanic communities, including acculturation issues,^8^ discrimination,^9^ and a lack of population-appropriate health services.

Access to firearms exacerbates suicide risk.^10–13^ Many people who attempt suicide ultimately survive^14^; however, survival is typically less likely for those who use firearms, given the 80%–90% case-fatality rate of firearm attempts.^15,16^ In 2020, firearms accounted for most suicide deaths in the United States^1^ and 40% of suicide deaths among Hispanic adults.^17^ Firearm suicide rates among Hispanic adults have increased by 42.9% since 2013,^1^ and recent data suggest that Hispanic adults purchased firearms at a 49% higher rate in 2020 than in 2019.^18^ The high lethality of firearm suicide attempts and the increase in firearm availability among Hispanic adults therefore poses a significant but ambiguous public health challenge.

Critically, there are gaps in characterizing firearm suicide risks and potential prevention opportunities in Hispanic communities.^5,19^ In particular, health services may be crucial for preventing firearm suicide deaths if Hispanic individuals at risk for suicide can be identified and offered effective, population-appropriate interventions,^20^ such as lethal-means counseling or psychotherapeutic and pharmacologic treatment.^21^ However, there is limited knowledge of whether Hispanic firearm suicide decedents are less likely than non-Hispanic firearm suicide decedents to interact with potentially life-saving mental health services prior to death.^22–24^ This is at least in part because much of the prior literature on firearm suicide death has focused on the high frequency of deaths among older, non-Hispanic White males (ie, the population historically experiencing the greatest burden of firearm suicide).

We sought to address these gaps and generate new insights on firearm suicide death among US Hispanic adults. Our primary research objective was to describe Hispanic adult firearm suicide decedents reported through the National Violent Death Reporting System (NVDRS) from 2013–2019, including their demographic characteristics, firearm choices, suicidal thoughts and behaviors, and mental health, compared with non-Hispanic adult firearm suicide decedents. Our secondary research objective was to examine the relationship between ethnicity and the likelihood of receiving mental health treatment among decedents in our sample. As a result, this study responds to the National Institutes of Health's (NIH’s) call for researchers to identify risk factors and improve suicide prevention in racial/ethnic minority communities and better understand firearm injury among health disparity populations,^25-27^ specifically focusing on Hispanic adults.

## Data and methods

### Data

We received de-identified data from the NVDRS Restricted Access Database (RAD)^28^ on all Hispanic adult firearm suicides from 2013–2019 through an approval process managed by the Centers for Disease Control and Prevention (CDC). The NVDRS program links data from vital records and investigative reports from coroners/medical examiners (CMEs) and law enforcement (LE) to compile comprehensive incident-level data on violent deaths from all 50 states, the District of Columbia, and Puerto Rico. The NVDRS contains demographic, mortality, circumstance, and weapon information, as well as unstructured CME and LE narratives summarized from interviews of persons close to each death. These records are linked to additional data sources, including toxicology information, Supplementary Homicide Reports, National Incident-Based Reporting System, medical information, and court records, where available.^29^

### Sample

We examined 82 226 adult firearm suicide cases recorded in NVDRS from 2013–2019. All decedents were identified using the NVDRS program's thorough process for determining suicide—which standardizes manner of death designations from death certificates and CME investigations across many different jurisdictions—and standardized information on the weapon resulting in each death (ie, firearm).^28^ Decedents ages 18 and older were included. Our cohort of interest included 3590 Hispanic adult firearm suicide decedents. The comparison group included 78 636 non-Hispanic adult firearm suicide decedents.

Not all states reported to NVDRS during our study period. Table S1 describes the states and years represented in the study sample. This study was deemed non–human subjects research using deidentified data on deceased persons only and did not require approval from the University of Utah Institutional Review Board.

### Variables

We divided the study sample into Hispanic and non-Hispanic firearm suicide decedents using the NVDRS ethnicity variable. The NVDRS follows US Department of Health and Human Services standards for race/ethnicity categorization. Decedents with Mexican, Puerto Rican, Cuban, Central or South American, or other Spanish culture or origin are coded as Hispanic, regardless of race.

We examined 1 continuous demographic variable (age) and 4 categorical demographic variables: sex, educational level, marital status, and veteran status. We examined these variables because previous studies have demonstrated that firearm suicide rates or firearm ownership tend to vary by age,^30,31^ sex,^32,33^ marital status,^34,35^ and veteran status^35,36^; however, little is known about these characteristics among Hispanic firearm suicide decedents specifically. We also examined the type of firearm involved in each death, organized into 5 categories (handgun, shotgun, rifle, submachine gun, and other), and birthplace (foreign- or US-born). Binary variables indicating a history of suicide attempt and a history of suicidal thoughts/plans before the fatal incident were also examined. Disclosure of suicidal thoughts/plans could be verbal, written, or electronic.

Last, we examined 3 binary variables related to mental ill health and mental health treatment prior to firearm suicide. These variables are endorsed by trained abstractors at the state level using the information provided in the CME and LE investigation reports and accompanying documents (eg, medical data), which, depending on the state, may or may not be directly included in the investigation reports. The first of these variables measured whether the decedent experienced a mental health or psychiatric problem prior to suicide, whether or not the condition directly contributed to the death. The variable equaled 1 if there was any indication of a mental health or psychiatric problem in the CME or LE investigation reports and accompanying documents provided to the abstractors, and it equaled 0 if not. Examples of disorders qualifying as mental health problems include diagnoses such as major depressive disorder or other mood disorders, schizophrenia, generalized anxiety or other anxiety disorder, neurodevelopmental disorders, eating disorders, personality disorders, and organic mental disorders (such as Alzheimer's).

The second variable was equal to 1 if the decedent was known to be undergoing treatment for a mental health or psychiatric problem at the time of death and 0 if not. The third variable was equal to 1 if the decedent had a known history of ever being treated for a mental health or psychiatric problem and 0 if not. Examples of evidence from the CME and LE investigation reports and accompanying documents provided to the abstractors indicating mental health treatment included seeing a psychiatrist, psychologist, medical doctor, therapist, or other counselor for a mental health condition; having a valid antidepressant or other psychiatric medicine prescription; or residing in an inpatient or group home facility for a mental health or psychiatric problem.

Data were non-missing for the mental ill health, mental health treatment, and suicidal thoughts and attempt variables. Data were missing for the demographic variables (a maximum of 6.94% were missing for educational level, and a minimum of 0.35% were missing for ethnicity). We used multiple imputation by chained equations to replace missing data with predictors containing non-missing observations.

### Analyses

To achieve the primary objective of this population-based observational study, we summarized each of our study variables for all decedents in the sample. We used bivariate *t* tests and chi-square tests to compare the Hispanic and non-Hispanic firearm suicide groups. Given the uncertainty in understanding whether Hispanic suicide decedents are less likely than their non-Hispanic peers to interact with mental health services prior to death, we also estimated logistic regression models to examine the relationship between ethnicity and the likelihood of receiving mental health treatment prior to firearm suicide.

Models 1 and 3 show the unadjusted odds of undergoing treatment for a mental health or substance use problem at the time of death and ever being treated for a mental health or substance use problem, respectively. To account for potential confounding factors, models 2 and 4 adjusted for age, sex, educational level, marital status, military veteran status, known history of suicidal thoughts and suicide attempts, and whether the decedent had a known mental health or substance use problem at death. Models 2 and 4 also adjusted for the US Census division in which the decedent lived to account for regional differences in the prevalence of firearm ownership, firearm suicide death, and mental health treatment capacity,^37,38^ and year fixed effects. Standard errors were clustered at the US Census division level. For ease of interpretation, we present the logistic regression model coefficients as odds ratios. An a priori significance level of .05 was established. All analyses were conducted using Stata MP version 17.1 (StataCorp, College Station, TX).

## Results

Hispanic and non-Hispanic adult firearm suicide decedents differed by all demographic variables described in Table 1. On average, Hispanic adults who died by firearm suicide were more likely to be younger (38.8 vs 51.2 years) and less likely to have served in the military (15.4% vs 25.0%) than non-Hispanic decedents (*P* < .001). Hispanic firearm suicide decedents were more likely to have never been married than non-Hispanic decedents (46.5% vs 28.7%; *P* < .001) but less likely to have had a bachelor's degree or higher educational level (9.3% vs 17.9%; *P* < .001). Table 2 describes whether decedents were foreign- or US-born. Approximately 17.6% of Hispanic decedents were born outside of the United States, compared to less than 3% of non-Hispanic firearm suicide decedents (*P* < .001).

**Table 1.** Describing the study sample (n = 82 226): 2013–2019.

	Hispanic (n = 3590)		Non-Hispanic (n = 78 636)		
	Mean/Frequency	Standard Deviation/Percent	Mean/Frequency	Standard Deviation/Percent	*P*
Age in years, mean, SD	38.8	16.6	51.2	19.0	<.001
Sex					<.001
Male	3194	89.0%	67 954	86.4%	
Female	396	11.0%	10 682	13.6%	
Military veteran					<.001
No	3038	84.6%	58 944	75.0%	
Yes	552	15.4%	19 692	25.0%	
Marital status					<.001
Married/civil union/domestic partnership	1156	32.2%	29 554	37.6%	
Never married	1668	46.5%	22 542	28.7%	
Widowed	93	2.6%	6056	7.7%	
Divorced	503	14.0%	17 057	21.7%	
Married/civil union/domestic partnership, but separated	109	3.0%	2292	2.9%	
Single, not otherwise specified	61	1.7%	1135	1.4%	
Educational level					<.001
8th grade or less	300	8.4%	2107	2.7%	
9th to 12th grade, no diploma	589	16.4%	7604	9.7%	
High school graduate or GED completed	1418	39.5%	33 453	42.5%	
Some college credit, but no degree	683	19.0%	14 385	18.3%	
Associate's degree	267	7.4%	7056	9.0%	
Bachelor's degree	235	6.5%	9520	12.1%	
Master's degree	74	2.1%	3108	4.0%	
Doctorate or professional degree	24	0.7%	1403	1.8%	

Authors’ analysis of National Violent Death Reporting System (NVDRS) Restricted Access Database (RAD) data.

**Table 2.** Describing the study sample's place of birth by decedent ethnicity (n = 82 226): 2013–2019.

	Hispanic (n = 3590)		Non-Hispanic (n = 78 636)		
	Frequency	Percent	Frequency	Percent	*P*
Foreign-born	632	17.6%	2109	2.7%	<.001
US-born	2958	82.4%	76 527	97.3%	

Authors’ analysis of National Violent Death Reporting System (NVDRS) Restricted Access Database (RAD) data. The foreign-born category included decedents born in Canada, Cuba, Mexico, and the “remainder of the world” categories documented in NVDRS.

Handguns historically account for most firearm suicide deaths.^39^ As shown in Table S2, handguns were used in three-fourths (74.9%) of non-Hispanic firearm suicide deaths but more than 8 in 10 (81.9%) Hispanic firearm suicide deaths (*P* < .001). Hispanic decedents were less likely than non-Hispanic decedents to use shotguns (7.5% vs 12.5%) or rifles (10.1% vs 12.0%) (*P* < .001).

Table 3 describes the history of suicidal thoughts and attempts, mental ill health, and mental health treatment prior to firearm suicide by decedent ethnicity. Compared with non-Hispanic decedents, Hispanic decedents were more likely to have a known history of suicidal thoughts/plans (31.1% vs 28.1%; *P* < .001), suicide attempt (13.6% vs 10.5%; *P* < .001), and both suicidal thoughts/plans and suicide attempt (8.7% vs 5.9%: *P* < .001). Hispanic decedents were more likely to have a known history of suicidal attempt without a history of suicidal thoughts/plans (ie, history of suicide attempt only; 5.9% vs 4.6%), but the difference was not statistically significant (*P* = .498). Hispanic firearm suicide decedents were also less likely to have a known mental health or psychiatric problem at the time of death (30.1% vs 37.5%: *P* < .001). Only 13.8% of Hispanic firearm suicide decedents were known to be undergoing treatment for a mental health or substance use problem prior to death, compared to 18.8% of non-Hispanic firearm suicide decedents (*P* < .001). Hispanic decedents were less likely than non-Hispanic decedents known to have ever been treated for a mental health or substance use problem (20.6% vs 25.9%; *P* < .001).

**Table 3.** Describing the study sample's known history of suicidal thoughts, behaviors, mental health, and mental health treatment by decedent ethnicity (n = 82 226): 2013–2019.

	Hispanic (n = 3590)		Non-Hispanic (n = 78 636)		
	Frequency	Percent	Frequency	Percent	*P*
Suicidal thoughts and behaviors					
Known history of attempting suicide					<.001
No/not available	3101	86.4%	70 369	89.5%	
Yes	489	13.6%	8267	10.5%	
Known history of suicidal thoughts or plans					<.001
No/not available	2475	68.9%	56 546	71.9%	
Yes	1115	31.1%	22 090	28.1%	
Known history of attempting suicide only (ie, no history of suicidal thoughts or plans)					.498
No/not available	3415	95.1%	74 994	95.4%	
Yes	175	5.9%	3642	4.6%	
Known history of suicidal thoughts or plans only (ie, no history of attempting suicide)					.886
No/not available	2789	77.7%	61 171	77.8%	
Yes	801	22.3%	17 465	22.2%	
Known history of both suicidal thoughts/plans and attempting suicide					<.001
No/not available	3276	91.3%	74 011	94.1%	
Yes	314	8.7%	4625	5.9%	
Mental health					
Known mental health or psychiatric problem at the time of death					<.001
No/not available	2789	69.9%	49 147	62.5%	
Yes	801	30.1%	29 489	37.5%	
Known to be undergoing treatment for a mental health or substance use problem at the time of death					<.001
No/not available	3093	86.2%	63 815	81.2%	
Yes	497	13.8%	14 821	18.8%	
Known history of ever being treated for a mental health or substance use problem					<.001
No/not available	2850	79.4%	58 233	74.1%	
Yes	740	20.6%	20 403	25.9%	

Authors’ analysis of National Violent Death Reporting System (NVDRS) Restricted Access Database (RAD) data.

Table 4 shows the results of our logistic regression analyses. On average, Hispanic firearm suicide decedents had 16.0% lower odds than non-Hispanic firearm suicide decedents of having been knowingly treated for a mental health or substance use problem prior to death, after adjusting for potential confounding factors (adjusted odds ratio [aOR] = 0.84; *P* = .005; model 2). Moreover, with all else equal, Hispanic firearm suicide decedents had a 22.0% lower adjusted odds than non-Hispanic firearm suicide decedents of having ever been knowingly treated for a mental health or substance use problem (aOR = 0.78; *P* < .001; model 4).

**Table 4.** Odds ratios from logistic regression models examining the relationship between ethnicity and undergoing mental health treatment among firearm suicide decedents (n = 82 226): 2013–2019.

Outcome	Known to be undergoing treatment for a mental health or substance use problem at the time of death		Known history of ever being treated for a mental health or substance use problem	
	1	2	3	4
	OR (95% CI)	aOR (95% CI)	OR (95% CI)	aOR (95% CI)
Ethnicity				
Non-Hispanic	Ref	Ref	Ref	Ref
Hispanic	0.70** (0.60, 0.81)	0.84** (0.75, 0.95)	0.75** (0.66, 0.87)	0.78** (0.68, 0.88)

Abbreviations: aOR, adjusted odds ratio; OR, odds ratio; Ref, reference.

Authors’ analysis of National Violent Death Reporting System (NVDRS) Restricted Access Database (RAD) data. We established an a priori significance level of .05. **Statistically significant difference at a level <.01. All models included 82 226 decedents. The ORs are estimates from bivariate logistic regression models (models 1 and 3). The aORs are estimates from multivariable logistic regression models (models 2 and 4), which adjusted for decedent's age, sex, educational level, marital status, military veteran status, history of suicidal thoughts, history of suicide attempts, whether the decedent had a mental health or substance use problem at death, US Census division, and year fixed effects. The full regression coefficients are provided in Table S3.

## Discussion

Although firearm suicide rates are rising among Hispanic adults in the United States, findings from our population-based study may help clinicians, public health practitioners, and policymakers better understand firearm suicide among Hispanic community members. By examining all adult firearm suicide deaths reported in NVDRS from 2013–2019, we were able to demonstrate key differences in demographic characteristics, firearm choices, and known history of suicidal thoughts and behaviors, mental ill health, and mental health treatment between Hispanic and non-Hispanic decedents. The high lethality of firearm suicide attempts poses an ongoing challenge for public health practitioners, clinicians, and Hispanic communities, although our findings may help inform additional research on opportunities for preventing Hispanic adult firearm suicide in clinical or community settings.

One key finding from our study was that Hispanic adult firearm suicide decedents were significantly less likely than non-Hispanic adult firearm suicide decedents known to be undergoing treatment for mental health or substance use problems prior to death, even after adjusting for the presence of mental ill health and other potential confounding factors. On the one hand, this finding is sobering but not surprising. Other studies have demonstrated that Hispanic persons are less likely than non-Hispanic persons to access mental health care,^22^ even when psychiatric illness is present, due to cultural stigma, limited health care options, legal concerns, and language or literacy barriers.^40–43^ Other studies have cited gender roles and *machista* attitudes as preventing Hispanic adults from seeking help for mental health.^44^ Regardless of the cause, mental health care disparities are specifically problematic in the context of firearm suicide because psychotherapy and pharmacologic therapy can help prevent suicide death,^21^ but the 80%–90% case-fatality rate of firearm suicide attempts often limits intervention opportunities once an individual has acted to end their life.

On the other hand, this finding may indicate important opportunities for health care and public health agencies. First, ensuring adequate care for Hispanic adults with psychiatric conditions may help save lives. Only 13.8% of Hispanic firearm suicide decedents in this study were known to be undergoing treatment for a mental health or substance use problem prior to death, although approximately 1 in 3 Hispanic decedents had a known mental health or substance use problem. This likely indicates an imperative for public health agencies and policymakers to make progress on initiatives that integrate mental health screening into primary medical care settings, reduce mental health stigma among Hispanics,^45^ and expand mental health treatment capacity in Hispanic communities (eg, reducing long lag times between implementing policy solutions for enhancing training programs and growing the mental health workforce).^46^ This is especially important for individuals who may have access to firearms, as greater mental health treatment capacity is associated with lower firearm suicide rates.^47^

Second, previous studies suggest that lethal-means assessment and counseling interventions may reduce firearm suicide attempts and improve opportunities to treat underlying health needs.^21,48–50^ However, lethal-means assessment and counseling interventions are infrequently used with Hispanic patients.^51^ One such intervention—Counseling on Access to Lethal Means (CALM)—incorporates counseling strategies for health professionals to help individuals and their families reduce access to firearms and other lethal means in times of crisis, including dialogue on safe firearm storage in the home.^52^ Because Hispanic individuals frequently turn to medical doctors for mental health concerns,^44^ and considering CALM can be provided in a range of settings, opportunities exist for local health care systems to adopt CALM into clinical practice and provide CALM training to mental health and non–mental health professionals alike. To support these efforts, policymakers and state health departments can help support, host, or promote training programs for CALM (eg, as exemplified in Utah^53^) and other lethal-means assessment interventions for local health system and community organizations, such as *Botanicas* and the *Ventanilla de Salud* program administered through Mexican consulates.^54,55^ Investing in lethal-means assessment in settings where Hispanics receive health care advice and treatment may be critical given the recent increase in Hispanic firearm ownership and because firearm suicide deaths are not exclusively driven by mental health.^56–59^

In addition to generating new insights on Hispanic adult firearm suicide decedents’ use of mental health care, we also described differences in suicidal thoughts and behaviors and firearm type between Hispanic and non-Hispanic decedents. For example, we found that Hispanic adult firearm suicide decedents were 29.5% more likely than non-Hispanic adult firearm suicide decedents to have a known prior suicide attempt. However, in narrowing this analysis, Hispanic decedents were not significantly more likely than non-Hispanic decedents to have a known history of attempting suicide without a history of suicidal thoughts/plans. We also demonstrated that Hispanic adult firearm suicide deaths were 9.3% more likely than non-Hispanic deaths to involve handguns. Collectively, these findings may be relevant for understanding Hispanic adults’ capability for suicide.^60^ According to theories of suicidal behavior aligned with the Intent-to-Action Framework,^60,61^ the capability for suicide is influenced by contributors that facilitate an individual to attempt suicide, including *practical contributors* such as those that increase knowledge of and access to lethal means. These distinctions may indicate key insights and motivate future studies to better inform when lethal-means counseling and other interventions should be offered to Hispanic individuals, considering factors contributing to individuals’ capability for suicide.

This study is among the first to describe important differences between Hispanic and non-Hispanic adult firearm suicide decedents. We found that Hispanic adults in our study sample were 38.4% less likely than non-Hispanic decedents to have been military veterans, on average. Military veterans experience elevated suicide risk^62^ and are often exposed to factors linked to suicide, such as posttraumatic stress disorder and access to lethal means,^62–64^ although non-veteran status does not preclude someone from experiencing suicide risk. We also found that fewer Hispanic decedents were divorced (14.0%) than non-Hispanic decedents (21.7%); relatively fewer Hispanic decedents attained at least a bachelor's degree (9.3% vs 17.9%). Although the relationship between educational attainment and suicide is complicated and likely varies by population, recent evidence demonstrating a negative relationship between having at least a college degree and suicide rates may suggest additional risk for Hispanic firearm suicide decedents who have lower educational attainment.^65^ Among Hispanics specifically, having a college degree may improve an individual's understanding of mental health conditions and the likelihood of receiving psychiatric treatment.^66^

Finally, 17.6% of Hispanic adult firearm suicide decedents in our study were born outside of the United States. Hispanic suicide risk varies between US- and foreign-born populations.^5^ Acculturation stresses and discrimination have also been shown to increase the risk of suicidal ideation and suicide attempts among Hispanics and immigrants.^9,67,68^ Additional data and research will enable the scientific community to investigate the burden of suicide and identifying critical suicide risk factors among Hispanic immigrants and different subpopulations (eg, Cubans, Puerto Ricans, Mexicans), especially research on multilingual, culturally appropriate services.

### Limitations

This study had several limitations. First, the findings described in this paper are descriptive, and we only examined decedent data. Understanding differences in the distributions of characteristics between Hispanic adult firearm suicide decedents and living Hispanic individuals may better inform opportunities for suicide prevention. For example, over the length of our study, it has been estimated that there are slightly more Hispanic males than females aged 18 years and older in the United States (50.6% male in 2013 and 50.3% in 2019).^69,70^ Yet, Hispanic adult firearm suicide decedents in our sample were predominantly male (89%). Moreover, in 2019, an estimated 16.8% of Hispanic adults aged 18 and older in the United States attained a bachelor's degree or higher,^71^ an increase from the estimated 13.1% of Hispanic adults aged 18 and older at the beginning of our study.^72^ Only 9.3% of Hispanic adult firearm suicide decedents in our sample attained a bachelor's degree or higher. Thus, our findings should not be used to predict or prevent suicidal or health care–seeking behavior in living populations. We only sought to describe and compare differences between Hispanic and non-Hispanic adult firearm suicide decedents, although additional studies comparing living individuals may yield insights on risk factors or prevention pathways.

Second, our analyses were not causal. Identifying causal links was not our intent. Rather, our results should be helpful for public health surveillance and informing additional studies related to intervention pathways.

Third, NVDRS abstractors endorse the variables on mental health and suicidal thoughts and attempts from CME/LE investigation reports. This information is often derived from interviews with next-of-kin. Although next-of-kin interviews may be subject to recall bias, NVDRS abstractors also commonly have the opportunity to receive information and endorse these variables from other documents (eg, medical data), whether included with or in addition to the investigation reports. Still, it is possible that mental health or treatment characteristics may have eluded the NVDRS abstraction process; it is unknown if any such occurrences would have differed by decedent ethnicity. Similarly, previous studies have demonstrated racial/ethnic differences in the length and choice of language used in the CME and LE narratives.^73^ These differences may create biases in endorsing or result in the misclassification of mental health treatment variables for Hispanic decedents.

Fourth, the earlier years of this study had fewer reporting states, which increased to 44 states and territories in 2019, limiting the generalizability of our findings.

Fifth, suicide may be underreported due to stigma and the misclassification of Hispanic ethnicity on death records, although the NVDRS program uses multiple data sources to improve ethnicity classification.

## Conclusion

This study responds to the NIH's call for researchers to identify risk factors and improve suicide prevention in racial/ethnic minority communities and better understand firearm injury among health disparity populations,^25–27^ specifically focusing on Hispanic adults. We examined all adult firearm suicide deaths reported through NVDRS from 2013–2019 and demonstrated key differences between Hispanic and non-Hispanic decedents. Hispanic adult firearm suicide decedents were less likely than non-Hispanic adult firearm suicide decedents known to be undergoing treatment for mental health or substance use problems before death. Hispanic adult firearm suicide decedents were also less likely than non-Hispanic decedents to have been military veterans, more likely to have had a known previous suicide attempt, and more likely to use handguns to take their own lives. These differences may indicate opportunities for policymakers and public health agencies to promote initiatives integrating mental health screening into primary medical care, reducing mental health stigma, and expanding mental health treatment capacity and lethal-means counseling in Hispanic communities.

## Supplementary Material

qxad016_Supplementary_Data
