# Supplementary material for: Critical gaps in understanding firearm suicide in Hispanic communities: demographics, mental health, and access to care
Source: Health Aff Sch. 2023 Jun 20;1(1):qxad016. doi: 10.1093/haschl/qxad016 (PMC11103729; doi:10.1093/haschl/qxad016)
Supplement: qxad016_Supplementary_Data [file qxad016_Supplementary_Data.zip › HAS - Supplementary Material - Goldstein et al - Correct.docx]

**Supplementary Material**

Table S1: States and years represented in the study sample from NVDRS

| **State** | **Years** |
| --- | --- |
| Alabama | 2018-2019 |
| Alaska | 2013-2019 |
| Arizona | 2015-2019 |
| California | 2017-2019 |
| Colorado | 2013-2019 |
| Connecticut | 2015-2019 |
| Delaware | 2017-2019 |
| District of Columbia | 2017-2019 |
| Georgia | 2013-2019 |
| Hawaii | 2015-2016, 2019 |
| Illinois | 2016-2019 |
| Indiana | 2016-2019 |
| Iowa | 2016-2019 |
| Kansas | 2015-2019 |
| Kentucky | 2013-2019 |
| Louisiana | 2018-2019 |
| Maine | 2015-2019 |
| Maryland | 2013-2019 |
| Massachusetts | 2013-2019 |
| Michigan | 2014-2019 |
| Minnesota | 2015-2019 |
| Missouri | 2018-2019 |
| Montana | 2019 |
| Nebraska | 2018-2019 |
| Nevada | 2017-2019 |
| New Hampshire | 2015-2019 |
| New Jersey | 2013-2019 |
| New Mexico | 2013-2019 |
| New York | 2015-2019 |
| North Carolina | 2013-2019 |
| Ohio | 2013-2019 |
| Oklahoma | 2013-2019 |
| Oregon | 2013-2019 |
| Pennsylvania | 2016-2019 |
| Puerto Rico | 2017-2019 |
| Rhode Island | 2013-2019 |
| South Carolina | 2013-2019 |
| Utah | 2013-2019 |
| Vermont | 2015-2019 |
| Virginia | 2013-2019 |
| Washington | 2016-2019 |
| West Virginia | 2017-2019 |
| Wisconsin | 2013-2019 |

Table S2. Describing the types of firearms used by decedents in the study sample: 2013-2019.

|  | **Hispanic (n = 3,590)** | | **Non-Hispanic (n = 78,636)** | |  |
| --- | --- | --- | --- | --- | --- |
|  | **Frequency** | **Percent** | **Frequency** | **Percent** | **P value** |
| Firearm type |  |  |  |  |  |
| Handgun (bolt-action, derringer, semi-automatic, and single-shot pistols; revolvers; and other) | 2,940 | 81.9% | 58,898 | 74.9% | <0.001 |
| Shotgun (automatic, bolt-action, double-barrel, pump-action, semi-automatic, single-shot, rifle combination, and other) | 269 | 7.5% | 9,830 | 12.5% |  |
| Rifle (automatic, bolt-action, lever-action, semi-automatic, single-shot, and other) | 363 | 10.1% | 9,436 | 12.0% |  |
| Submachine Gun or Other (e.g., handmade gun) | 18 | 0.5% | 472 | 0.6% |  |
| **Observations** | 82,226 |  |  |  |  |

Notes: Authors’ analysis of NVDRS RAD data. Firearm subtypes are shown in parentheses.

Table S3. Adjusted odds ratios from logistic regression models examining the relationship between ethnicity and undergoing mental health treatment among firearm suicide decedents (n = 82,226): 2013-2019.

|  | Outcome: Known to be undergoing treatment for a mental health or substance use problem at the time of death | Outcome: History of ever knowingly being treated for a mental health or substance use problem |
| --- | --- | --- |
| Ethnicity (Reference: Non-Hispanic) |  |  |
| Hispanic | 0.84** | 0.78** |
|  | 0.052 | 0.051 |
| Age of victim (years) | 1.00 | 0.99** |
|  | 0.005 | 0.002 |
| Sex (Reference: Male) |  |  |
| Female | 1.24** | 1.14** |
|  | 0.05 | 0.036 |
| Education Level (Reference: 8th grade or less) |  |  |
| 9th to 12th grade, no diploma | 0.92 | 1.05 |
|  | 0.097 | 0.078 |
| High school graduate or GED completed | 1.08 | 1.24** |
|  | 0.127 | 0.103 |
| Some college credit, but no degree | 1.16 | 1.36** |
|  | 0.121 | 0.075 |
| Associate's degree | 1.35* | 1.52** |
|  | 0.178 | 0.121 |
| Bachelor's degree | 1.31+ | 1.39** |
|  | 0.196 | 0.144 |
| Master's degree | 1.46** | 1.58** |
|  | 0.177 | 0.133 |
| Doctorate or Professional degree | 1.42* | 1.53** |
|  | 0.205 | 0.166 |
| Marital Status (Reference: Married/Civil Union/Domestic Partnership) |  |  |
| Never Married | 0.75** | 0.85** |
|  | 0.045 | 0.046 |
| Widowed | 0.71** | 0.79** |
|  | 0.05 | 0.057 |
| Divorced | 0.75** | 0.87** |
|  | 0.016 | 0.027 |
| Married/Civil Union/Domestic Partnership, but separated | 1.11 | 1.30* |
|  | 0.081 | 0.151 |
| Single, not otherwise specified | 0.75* | 0.76+ |
|  | 0.107 | 0.117 |
| Military Veteran (Reference: No) |  |  |
| Yes | 0.90** | 0.87** |
|  | 0.037 | 0.035 |
| Mental Health or Substance Use Problem at Time of Death (Reference: No) |  |  |
| Yes | 22.99** | 24.56** |
|  | 1.633 | 1.985 |
| Suicide Attempt History (Reference: No) |  |  |
| Yes | 1.19** | 1.74** |
|  | 0.05 | 0.08 |
| Suicide Thoughts History (Reference: No) |  |  |
| Yes | 1.36** | 1.68** |
|  | 0.104 | 0.112 |
| U.S. Census Division (Reference: Mountain) |  |  |
| East North Central | 0.92** | 0.75** |
|  | 0.008 | 0.007 |
| East South Central | 0.39** | 0.67** |
|  | 0.009 | 0.014 |
| Middle Atlantic | 1.25** | 1.00 |
|  | 0.014 | 0.014 |
| New England | 0.83** | 0.70** |
|  | 0.006 | 0.009 |
| Pacific | 0.73** | 0.77** |
|  | 0.009 | 0.013 |
| South Atlantic | 1.07** | 0.99 |
|  | 0.018 | 0.019 |
| West North Central | 0.83** | 0.78** |
|  | 0.017 | 0.019 |
| West South Central | 0.82** | 0.62** |
|  | 0.027 | 0.023 |
| Year (Reference: 2013) |  |  |
| 2014 | 0.66** | 0.69** |
|  | 0.035 | 0.088 |
| 2015 | 0.54** | 0.60** |
|  | 0.048 | 0.07 |
| 2016 | 0.55** | 0.65** |
|  | 0.043 | 0.085 |
| 2017 | 0.60** | 0.70** |
|  | 0.043 | 0.08 |
| 2018 | 0.55** | 0.67** |
|  | 0.049 | 0.088 |
| 2019 | 0.55** | 0.59** |
|  | 0.057 | 0.063 |
| **Observations** | 82,226 | 82,226 |

Notes: Authors’ analysis of NVDRS RAD data. We established an a priori significance level of 0.05. **P<0.01, *P<0.05, +P<0.10.
